# An Improved Indoor Positioning System Using RGB-D Cameras and Wireless Networks for Use in Complex Environments

**DOI:** 10.3390/s17102391

**Published:** 2017-10-20

**Authors:** Jaime Duque Domingo, Carlos Cerrada, Enrique Valero, Jose A. Cerrada

**Affiliations:** 1 Departamento de Ingeniería de Software y Sistemas Informáticos, ETSI Informática, UNED, C/Juan del Rosal, 16, 28040 Madrid, Spain; ccerrada@issi.uned.es (C.C.); jcerrada@issi.uned.es (J.A.C.); 2 School of Energy, Geoscience, Infrastructure and Society, Heriot-Watt University, Edinburgh EH14 4AS, UK; e.valero@hw.ac.uk

**Keywords:** indoor positioning, WPS, RGB-D sensors, WiFi, *fingerprinting*, trajectory, skeletons, *depth map*, IPS, Kinect

## Abstract

This work presents an *Indoor Positioning System* to estimate the location of people navigating in complex indoor environments. The developed technique combines *WiFi Positioning Systems* and *depth maps*, delivering promising results in complex inhabited environments, consisting of various connected rooms, where people are freely moving. This is a non-intrusive system in which personal information about subjects is not needed and, although RGB-D cameras are installed in the sensing area, users are only required to carry their smart-phones. In this article, the methods developed to combine the above-mentioned technologies and the experiments performed to test the system are detailed. The obtained results show a significant improvement in terms of accuracy and performance with respect to previous WiFi-based solutions as well as an extension in the range of operation.

## 1. Introduction

*Indoor Positioning Systems* (IPSs) are techniques employed to calculate the position of people or objects inside buildings. Among active localization techniques, and due to the extended use of WiFi devices (i.e., cellphones) as well as infrastructure (i.e., routers), WiFi-based positioning systems (WPSs) are a suitable opportunity to track the movements of people in indoor environments. However, these techniques do not deliver very precise results as stated in [1]. Aiming to solve this issue, other positioning techniques can be additionally used to provide more reliable results. A possible solution, based on computer vision, is the use of RGB-D cameras. This passive technique has been rapidly developed in the last few years, delivering promising outcomes for people identification [2], positioning [3,4,5] and gesture recognition [6].

The combination of the above-mentioned techniques, WPSs and RGB-D devices, has already been presented in a previous work [7]. In that approach, the WPS delivers the position of users in a range of meters and RGB-D cameras refine this value to a precision of centimeters. Within this paper, a more robust and extended infrastructure is presented, applying the mentioned idea to real environments, such as housing facilities.

The article is structured as follows: Section 2 explores existing solutions regarding positioning, based on WPS, RGB-D sensors, and combining both technologies. Section 3 illustrates the proposed system. In Section 4, the different experiments carried out of people navigating indoors are reported. An overall discussion on the obtained results is stated in Section 5. Finally, Section 6 provides remarks on the advantages and limitations of the presented system and suggests future developments based on this method.

## 2. Overview of Related Work

As other navigation systems, IPSs lean on the calculation of the position of a user in the environments. This localization process can be arranged in four different groups, as stated by Fallah et al. in [8]: (1) *Dead-reckoning*, where position of users is obtained based on a previously estimated or known position through the use of sensors such as accelerometers, magnetometers, compasses, and gyroscopes or using a user’s walking pattern; (2) *direct sensing*, which determines the location of users through the sensing of identifiers or tags installed in the environment, such as infrared (IR), ultrasound (USID), Bluetooth beacons or bar-codes; (3) the third group uses *Triangulation* by means of infrared, ultrasound or *Radio Frequency Identification* (RFID); and finally (4), *Pattern recognition* uses data from one or more sensors carried by the user and compares the obtained data with prior collected data. Computer vision and *fingerprinting* are examples of pattern recognition.

A second classification is presented in [9], where IPSs are categorized according to the technology in which they rely on. Following this guideline, Koyuncu and Yang suggest four different groups: (1) IPSs based on infrared or ultrasonic distances; (2) systems based on *Received Signal Strength Indication* (RSSI) triangulation or *fingerprints* by means of *Wireless Local Area Network* (WLAN) or RFID technology; (3) systems based on computer vision or on the combination of technologies like RFID or WLAN and (4) inertial technologies (e.g., gyroscopes) or other types of sensors.

In the next subsections, some of these categories, on which the navigation system proposed in this paper is based, are detailed and some previous works are compared.

### 2.1. WiFi Positioning Systems

*WiFi Positioning Systems* are mostly founded on the *fingerprinting* technique [10]. The aim of this method is the generation of a map of the environment by means of the recorded RSSI around the evaluated area. RSSI is a reference scale used to measure the power level of signals received from a device on a wireless network (usually WiFi or mobile telephony). The produced map is employed to obtain the position of a user in real time, comparing the values received from the user’s portable device to those stored in the map.

A recent comparison between WiFi-*fingerprint*-based indoor positioning systems has been presented by He and Chan [11]. Regarding the use of advanced techniques, the authors explain how to make use of temporal or spatial signal patterns, user collaboration, and motion sensors. In addition, the authors discuss recent advances on reducing offline labor-intensive survey, adapting to *fingerprint* changes, calibrating heterogeneous devices for signal collection, and achieving energy efficiency for smart-phones.

Systems based on WiFi-*fingerprint* do not deliver accurate results in clutter indoor environments. As stated in [7], the usual WPS error of positioning people inside a room is 2 m. This value results in an inaccurate indoor positioning, despite the use of Inertial Measurement Units (IMUs).

### 2.2. Positioning Systems by Means of Computer Vision

Vision-based technologies are an alternative system to estimate the position of users in different kinds of environments. These solutions are simple and inexpensive, above all if the system only consists of one or a few cameras. A survey of optical indoor positioning system is presented in [12]. In this work, authors classify the existing alternatives according to the reference used to figure out the position of users in the scene (e.g., 3D models, images, coded markers or projected patterns).

In [13], normal surveillance cameras are used to capture video. From that video, the system extracts the foreground objects using a background model. After a 2D Direct Linear Transformation, the objects are displayed on a bird’s eye map. Although this system provides information about the user’s position, it is not able to obtain the user’s identification.

Among vision-based devices, it is worth mentioning RGB-D sensors, increasingly developed during the last years. Positioning systems, such as OpenPTrack [3], which creates a scalable, multi-camera solution, have been developed for tracking people. In addition, Ye et al. [14] developed a method to detect and identify several people that are occluded by others in a scene. Furthermore, a third approach to obtain the position of users, the method *Kinect Positioning System* (KPS), is analyzed in [5].

Providing a more robust solution, Saputra et al. [4] present an indoor human tracking application using two depth-cameras, and the work in [15] proposes a model for merging Kinect trajectories similar to the one used in this article. Considering the global coordinates at the same time stamp, the system is able to determine the distance between two skeletons in order to discern between different users. However, this solution does not retrieve data from any other device (i.e., smart-phones) and, therefore, it is not able to deliver the position of each user.

One of the main limitations of IPSs based on computer vision is the complexity of identifying users in the scenario. Therefore, systems have to differentiate users by means of additional techniques, such as real-time face detection [16], or by means of color or patterns code [2]. However, both techniques spark problems with occlusions and the need of directly involving users in the system.

Following a more complex technique, Werner et al. [17] developed a system, called DeepMoVIPS, which relies on images obtained by smart-phones from which several features are extracted by means of convolution and pooling and, subsequently, classified. The location process is carried out after a training period that will be affected by changes in the scenario. In addition, in [18], authors use a Microsoft Kinect (Redmond, WA, USA) carried by a person in order to calculate user’s trajectory. However, as in the above-mentioned approaches, these two last solutions require a direct involvement of users. Additionally, the presence of other users in the images or changes in the scenario can affect the result.

The method proposed in this paper makes use of the precision of computer vision systems, but in a ’transparent’ manner, where users do not directly interact with the system.

### 2.3. Combined Positioning Systems

As stated by numerous authors, the combination of multiple technologies improves the efficiency of traditional WPSs [11,19,20]. Hybrid IPSs combine WPS with other technologies like *Global System for Mobile communication* (GSM) [21], bluetooth [22], RFID [23], or other Inertial Navigation Systems (INS), i.e., compasses, gyroscopes, accelerometers, atmospheric pressure sensors [24].

From the point of view of computer vision, Mirowski et al. [25] analyze how to generate a WPS *fingerprint map* with an RGB-D sensor mounted on a robot. In addition, in [26], a robot is located by means of three different systems: a laser rangefinder, a depth camera and RSSI values. Each system is used independently, according to the zone where the robot is located. Nonetheless, as the previous work, this approach does not exploit the different technologies simultaneously. In the first work, RGB-D sensors are used to obtain the real position and, in the second work, authors only use one system at a time.

Combined systems offer more accurate results, but usually require special devices. As previously mentioned, a new solution for indoor positioning is detailed in this paper. Based on the approach presented in [7], two techniques of different natures (i.e., WPS and RGB-D sensors) have been combined in a unique system to improve the accuracy of previous works dealing with this issue. Regarding the solution previously presented, in this extended approach, experiments have been carried out in inhabited interiors by means of a broader infrastructure, delivering promising results as is shown in the following.

## 3. Analysis of the System

**Figure 1:**
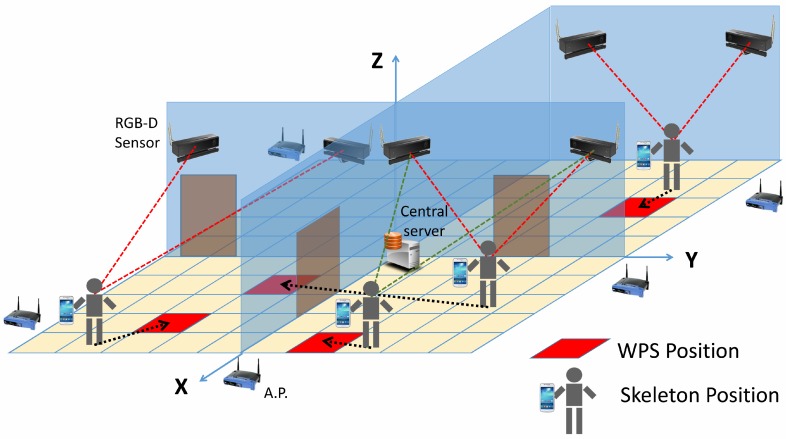
Scenario of the system.

The main objective of this system is to identify and estimate the position of users in complex inhabited scenarios, where several people are simultaneously navigating, by means of WPS and computer vision-based technologies. Both technologies, WPSs and depth cameras, are connected to a central server as shown in Figure 1. This central server is responsible for obtaining the required data and carrying out positioning calculations. The WPS makes use of several Access Points (APs) located around the environment (i.e., routers), which are within reach of the users’ cellphones. Cellphones measure the level of the received signals and send data to a web server. Simultaneously, each RGB-D sensor records a part of the scenario, by means of a camera and an infrared projection system, obtaining depth maps. These are processed to identify the skeletons of people and to refine the results delivered by the WPS, providing more accurate values for positioning. Finally, the central server obtains the positions of people and returns the calculated values to the cellphones.

Note that skeletons are obtained by means of the techniques presented in [27,28], where authors propose new algorithms to quickly and accurately predict 3D positions of body joints from depth images. Those methods form a core component of the Kinect gaming platform.

The working process of the developed system is divided into two main stages: *Training* and *Operational*. These stages are explained in the following subsections.

### 3.1. Training Stage

**Figure 2:**
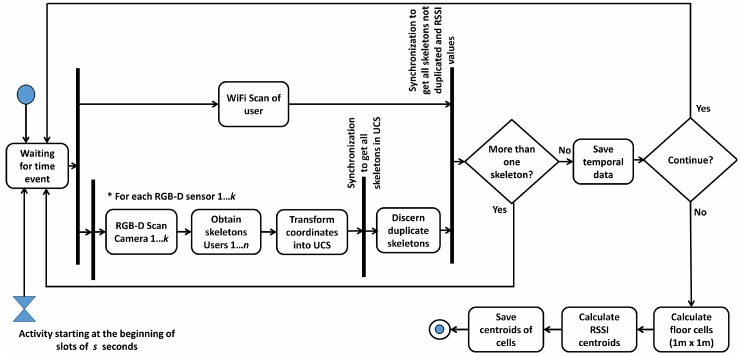
Activity diagram during the Training stage.

During the Training Stage, a user freely moves around the environment. Figure 2 shows, with an activity diagram, the different steps during this process. RSSI values from the cellphones, together with their position estimated by the depth sensors, are recorded on a *fingerprint map*.

From depth images, body joints are extracted and coordinates corresponding to the neck $\left(x_{n},y_{n},z_{n}\right)$ are considered, as this is the joint less prone to be occluded by elements in the scenario. In addition, a general universal coordinate system (UCS) has been created for the full scenario.

During the *WiFi Scan*, portable devices obtain RSSI values for each AP and send the data to the server. Concurrently, the *RGB-D Scan* is started in each one of the *k* different sensors. This process returns the above-mentioned coordinates $\left(x_{n},y_{n},z_{n}\right)$ of the person detected in the room. These coordinates are transformed into the general UCS. If there are duplicated skeletons, obtained by two or more RGB-D sensors, they are not considered. Then, the system saves the data of RSSI and the position of the user from the *depth map*.

When the user stops the loop, aiming to simplify the positioning process without significant loss of precision, another essential task is carried out at the end of this stage: the complete scenario is divided in cells of 1 × 1 m and, RSSI data are grouped in each cell, using the cell position of the skeleton. An RSSI vector is created for each cell, pairing each component to the centroid for all of the RSSI measurements of a certain AP. RSSI scans are grouped according to the distance between their original associated skeleton and the coordinates of the center of each cell. This step reduces the size of the *fingerprint map* to improve the performance of the system and obtains more reliable RSSI values avoiding variations in measures.

Note that, in this stage, only one user is registered on the *fingerprint map* by each RGB-D camera at a time and, simultaneously, RSSI values obtained by their portable device and coordinates of their skeleton are stored. However, if various users are involved in the creation of the *fingerprint map*, the system records positioning data for each user whenever they are situated in different rooms. This implies that an RGB-D camera will not detect more than one skeleton, and, therefore, there will not be conflict between skeletons and WPS values. The system could also be trained using markers for more than one user. It could identify the skeleton of each user using, i.e., different color shirts. The work [2] tracks construction workers considering different color-coded construction hard hats as a key to differentiating personnel.

### 3.2. Operational Stage

**Figure 3:**
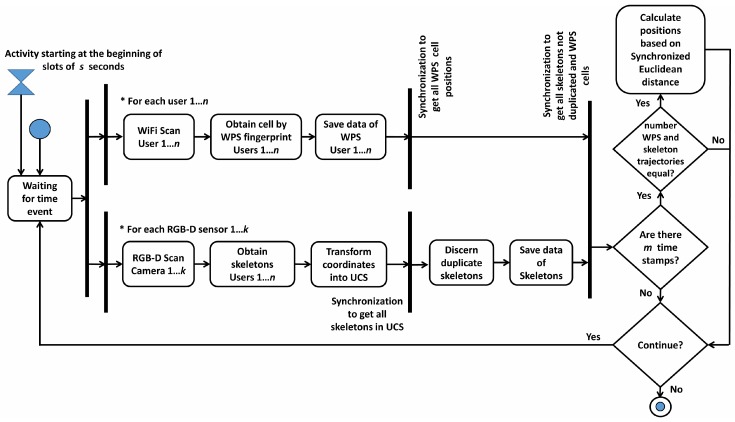
Activity diagram during the Operational stage.

This stage represents the usual working mode of the system. As shown in Figure 3, the process of obtaining the position of users is launched every *s* seconds. The system considers that *n* users are moving around the scenario and, synchronously, send their RSSI values to the central web server. Concurrently, the *RGB-D Scan* process is executed aiming to obtain the skeletons of people detected in the complete scenario. The coordinates returned by RGB-D sensors are transformed into the UCS, and duplicated skeletons obtained by two or more RGB-D sensors are discarded. Finally, after *m* time stamps, if the number of WPS and skeleton trajectories matches (i.e., equals *n*), the IPS estimates the position of each user. If this restriction is not satisfied, the system continues working until the number of trajectories matches.

**Figure 4:**
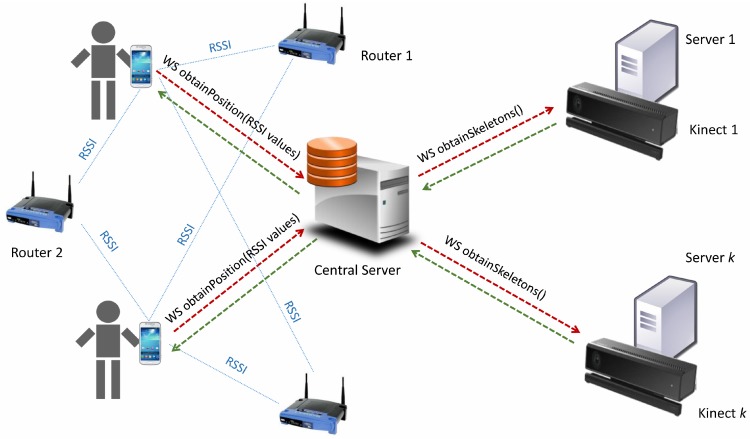
Scheme of web service callings.

As previously mentioned, each user carries a *smart-phone* with an app that synchronously and periodically sends its RSSI values to a central server through a web service. This web server hosts the complete database, recording the RSSI values. At the same time, this central server creates several threads for calling different web services, one for each RGB-D sensor, following the diagram in Figure 4. Concurrently, each RGB-D device is connected to a different web server, which sends the data to the central server. Furthermore, finally, this central server computes the result of the position merging WPS and RGB-D outcomes for each time stamp.

Note that the presence of several people moving in a large environment entails additional requirements. For example, the trajectories of users are tracked by means of more than one RGB-D sensor in some cases, obtaining a set of 3D user coordinates for each time stamp. These values (i.e., skeletons), which belong to the same person, are synthesized and transformed to the same coordinates system, as proposed in [29]. In case the Euclidean distance between several user coordinates, obtained by different sensors, is under a threshold (50 cm has been considered as the imaginary cylinder where people fit while they are standing up), only a user is considered to be in that position. Furthermore, if a user is detected by two or more RGB-D sensors, all of them track their movements and, thus, their trajectories. This is especially useful when people leave a room and another RGB-D identifies their movements.

When there is a fall in the system, the Operational stage restarts and new trajectories are obtained.

#### Position Matching Algorithm

**Figure 5:**
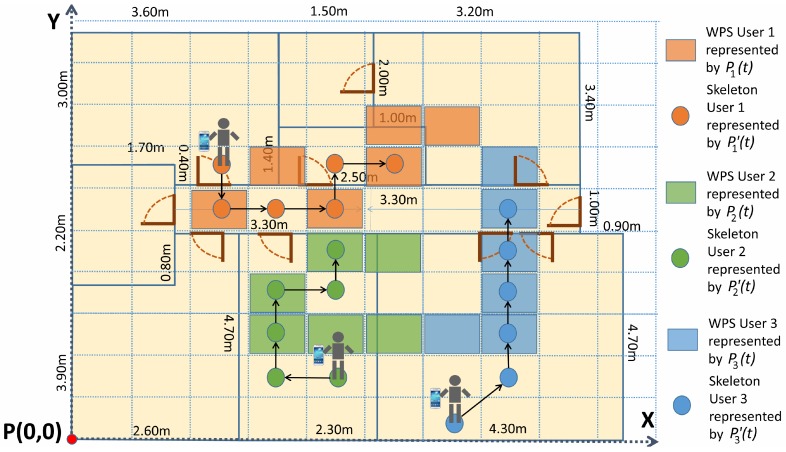
Cells with WPS and skeleton trajectories during the Operational stage.

As explained before, there are data, from both WPS and RGB-D devices, corresponding to several users that have been registered at the same time. It is necessary to properly match both kinds of data to deliver correct (and unique) positioning information for each user at each time stamp. The algorithm developed to solve this issue takes into account the following considerations:

- Let *n* be the number of users detected and *m* the number of time stamps registered in an experiment. Both positioning systems provide their corresponding position measurement for all of the users at each time stamp.
- Let $P_{i}\left(t\right)$ be the position of the user *i* provided by the WPS at the time stamp *t*, and let $P_{j}^{\prime }\left(t\right)$ be the skeleton position of the user *j* provided by the RGB-D sensors system at the time stamp *t*. It is important to remark that the users’ numbering of the WPS is not the same as that of the RGB-D sensors, which means that initially $P_{i}\left(t\right)$ is not associated to $P_{i}^{\prime }\left(t\right)$ but to another $P_{j}^{\prime }\left(t\right)$. The problem consists in determining, for all the time stamps considered, which one is the *i* index associated with every *j* index. Figure 5 shows an example of three different user trajectories with their corresponding paths of WPS and skeletons for six different time stamps.
- Let $s_{ij}$ be the binary element of the *Matching Matrix, S*, in the row *i*, column *j*. It links the trajectory of the user *i*, provided by the WPS, and the trajectory of the user *j*, provided by the RGB-D sensors system.
- The solution to the algorithm is to find the matrix *S*, valid for the complete dataset of the experiment, such that:
  $$s_{ij}/i=1\cdots n,j=1\cdots n\wedge s_{ij}\in \left\{0,1\right\}\wedge \sum_{x}s_{xj}=1\wedge \sum_{y}s_{iy}=1.$$

In order to find the matrix *S*, all the *m* time stamps considered must be taken into account, in such a way that the problem is seen as an optimization of the combination between WPS and skeleton trajectories for all the users. The best combination is defined as the one achieving the sum of distances among all possible users trajectories to be minimum, as shown in Equation (1):

(1) $$\begin{array}{c} \min\sum_{i,j}s_{ij}\times \sum_{t=1}^{m}d_{E}(P_{i}\left(t\right),P_{j}^{\prime }\left(t\right)), \end{array}$$

where $d_{E}$ represents the Euclidean distance between a skeleton and a WPS cell. This is measured as the distance between the coordinates of the neck of the skeleton and the centroid of the WPS cell. This expression (1) considers the *Synchronized Euclidean distance* [30], computing the sum of distances between each pair of points of WPS and skeleton trajectories, and it is solved using linear programming. The *Balas Additive Algorithm* [31] has been considered to solve the problem.

The *Synchronized Euclidean distance* is presented by [30] as a method to calculate the distance between sets of points at identical time stamps. If two trajectories with different points are obtained at the same time, for each pair of points, the total error is measured as the sum of the Euclidean distances between each pair of points at synchronized time stamps. This method allows for discerning what trajectory of a group is more similar to another one.

The achieved solution links each user to their skeleton positions in the UCS. Although some cells are located between two or more rooms, the system obtains the final position of the users according to Kinect sensors. It means that the system relates each WPS cell trajectory to the corresponding skeleton trajectory, but using the skeleton coordinates as the real user’s positions, and considering WPS information just for obtaining their identifications.

If two or more RGB-D sensors detect the same user, that user will be situated at the same position of the UCS (an error of 0.5 m between users is considered for discerning two users). One restriction to the model is the fact that if an RGB-D sensor has detected *z* skeletons, the results must conclude that there are *z* users in the range of that RGB-D sensor. The system might detect a user in the adjacent room if their position is near the wall and this restriction is not applied. This happens because the floor has been divided into cells in the general UCS, and some of these cells are, at the same time, in different rooms. Finally, the algorithm considers that the number of WPS trajectories must be equal to the number of skeleton trajectories.

## 4. Experimentation

**Figure 6:**
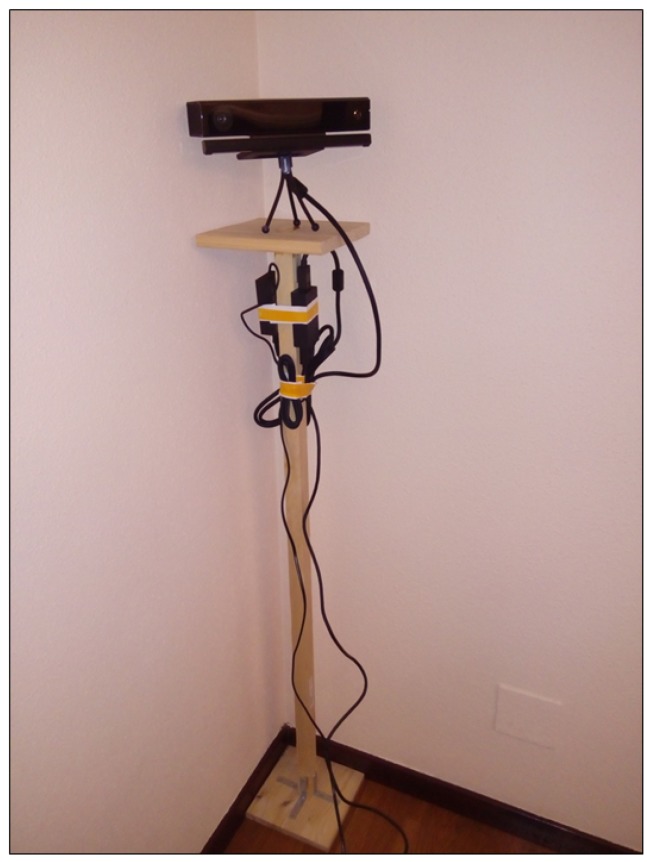
Kinect sensor mounted on a platform.

Kinect v2 sensors have been used to obtain depth information. These devices, based on Time of Flight (ToF) technology, deliver up to 2 MPixels images (1920 × 1080) at 30 Hz and 0.2 MPixels *depth maps* with a resolution of 512 × 424 pixels. The horizontal field of view of the RGB-D sensor is 70°, with a working range of 4.5 m. All of these devices, mounted on a tripod over a wooden platform (see Figure 6), have been connected to a web server where data are saved and processed.

It is worth mentioning that these sensors need a high bandwidth to transfer data, as they deliver 30 MB per second. As advised in [32], each device requires its own USB 3.0 hub. Using two or more devices connected to the same computer will demand extra PCI bus USB cards, as most current computers only have one USB 3.0 port (or two of them connected to the same hub).

**Figure 7:**
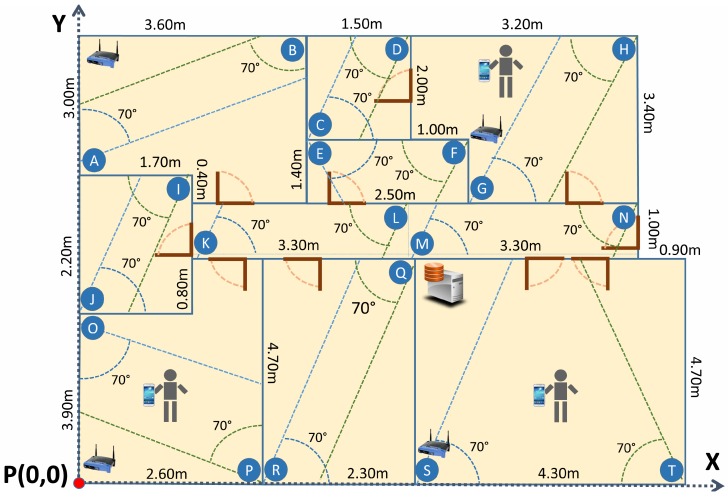
Plan of the office used in the experiments.

The experiments have been carried out in an 80 m^2^ office, with eight rooms and a central corridor, where 20 RGB-D cameras, Kinect v2, have been deployed covering all the accessible space (see Figure 7 where devices are represented by labeled circles).

Users have been equipped with Lenovo A936 smart-phones (Beijing, China), running on Android 4.4 Kitkat (Google Inc., Mountain View, CA, USA). An Apache Tomcat 8.0.27 server has been installed in the central server and in the servers connected to each RGB-D sensor.

**Figure 8:**
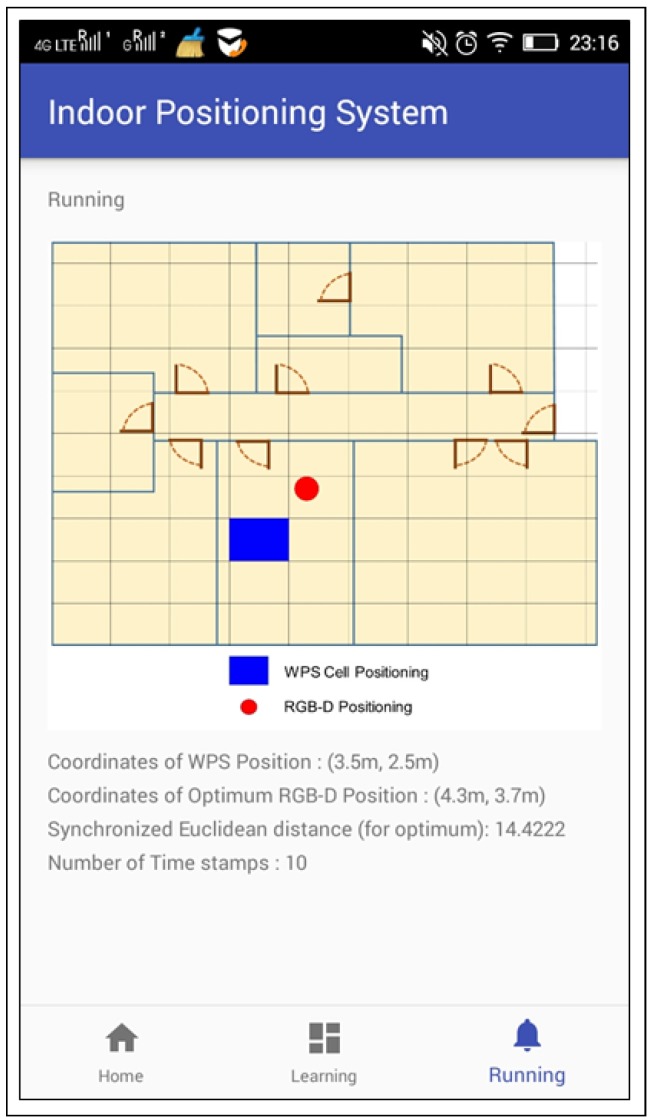
Screenshot of the application developed for Android devices.

Users’ smart-phones have established a connection to the local WiFi network. The smart-phones have obtained RSSI data and these were synchronously sent to the central web server, via SOAP protocol. At the same time, this central web server has started 20 different threads. Each thread has called a different web service to obtain the skeletons and each web service has been hosted in the respective server connected to the associated Kinect. When these threads have finished, the complete information has been saved and processed. An Android application, with Training and Operational mode, has been developed. It tracks the user’s position by means of WPS and the developed IPS (see Figure 8).

The developed application does not have special requirements and, any smart-phone with Android 4.0 or higher would be compatible.

Although all the experiments have been carried out in a LAN, the connection between the smart-phones and the central web server can also be performed by means of a wireless data network of telephony (3G, 4G, etc.).

**Figure 9:**
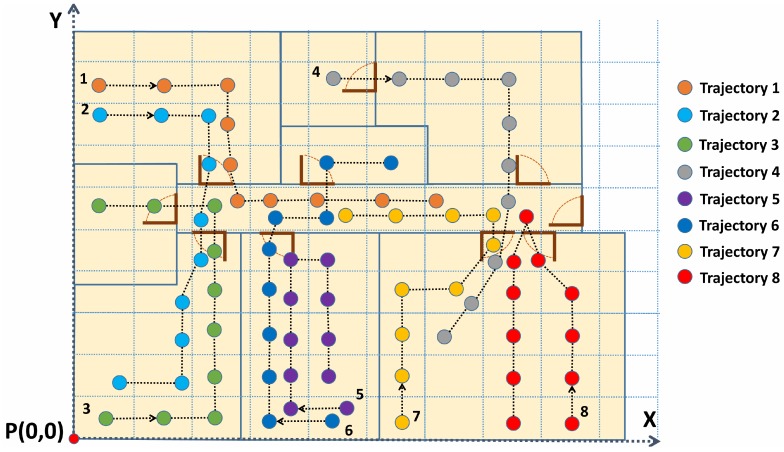
Some of the trajectories followed by users.

The performance of the system has been evaluated by a group of users going over 50 different trajectories. These trajectories have considered multiple types of movements. Some of the trajectories have started in a room and finished in another one. Other trajectories have just included movements in one room, or movements from one to another room, returning to the first one. Some paths have intersected or overlapped some parts of other ones. Others have simply been similar in path. As illustrated in Figure 9, ten time stamps have been recorded for each trajectory with an interval of 2 s between consecutive measurements.

The synchronization between the smart-phones and the central server has been done with an external time-synchronization service (*time.nist.gov*) by means of the network time protocol (NTP). Slots of 2 s between time stamps have been considered to obtain the RSSI measurements and skeletons. Each server connected to a Kinect synchronizes with the central server using an internal time-synchronization service. The central server acts as *domain controller* and represents the authoritative time-source for the rest of computers of the internal sub-network.

In order to evaluate the system for several users, the trajectories have been combined to complete more than one million of the possible random situations by a number of users. Up to 20 users have been simultaneously tracked in the experiments.

## 5. Results Discussion

The experiments have been carried out considering 10 consecutive time stamps (2 s). As depicted in Table 1, shorter experiments are not reliable to establish a comparison between RGB-D and WPS trajectories, especially when the number of users increases.

**Figure 10:**
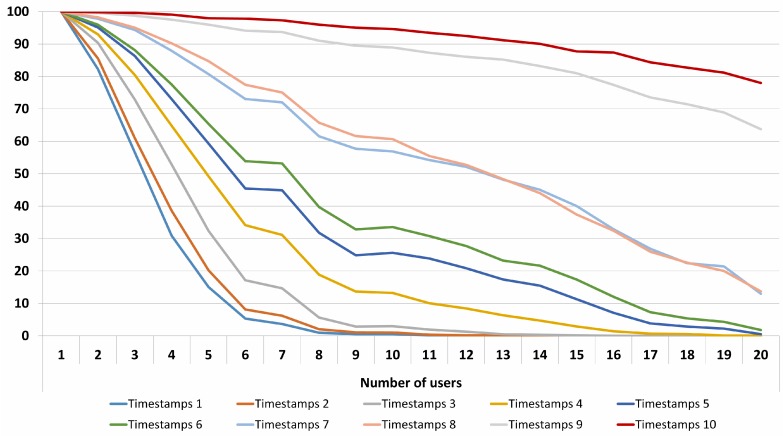
Positioning success (%) for a different number of users and time stamps.

The obtained results for 10 time stamps show an important improvement in terms of indoor positioning inside buildings. As expected, when the number of users increases, the success rate decreases. The system is able to precisely identify and locate up to 10 users with a success rate of 95%. For up to 15 users, the IPS has a success near 90%. In Figure 10, the results for different numbers of time stamps and users are displayed.

Other methods [11,19,20] refer to the positioning error as the distance error between the obtained user’s position and the real one. In this work, success is achieved when exact positions of all users are obtained correctly. This makes a comparison between the proposed method and other works complicated. However, considering an error of 0.2 m for users successfully detected and 2.0 m for the rest (WPS error obtained in the presented scenario), the average distance error is 0.29 m for 10 users and 0.59 m for 20 users (with 10 time stamps), as shown in Table 2.

Finally, computational cost of the system is mostly affected for the cost of obtaining depth maps and extracting the skeletons, and the cost of obtaining RSSI values. These two operations take tenths of a second. The rest of the operations are executed in milliseconds.

## 6. Conclusions

This work presents a method for indoor positioning in complex environments. It is based on a previously developed algorithm that worked for reduced spaces (i.e., one room). This new method facilitates indoor positioning in a complete floor or building. The combination of wireless networks with RGB-D sensors is a simple and economical method to increase the performance of WPS in interiors. The developed experiments allow locating more than 10 people with a success over 95% in a scenario with multiple rooms.

According to the taxonomy established by [33], the developed system obtains physical absolute position, operates recognizing people and processes data in remote servers. Its precision, cost and limitations are defined by the used RGB-D sensors. Regarding scaling, the system is prepared to work on large floors or in large environments.

The WPS position has not considered other INS, like compasses, gyroscopes or accelerometers [24]. The use of these sensors could improve the WPS positioning and, likely, the presented IPS. Because this system is based on the similarity between WPS and RGB-D trajectories, if WPS trajectories are enhanced with these sensors, they would be more similar to RGB-D ones and, hence, the system could be improved. Future research will consider the use of these sensors with the IPS.

The increase of APs improves the WPS positioning. In scenarios with a WPS error over 2 m, the use of more APs result in a more robust *fingerprint map*, and, therefore, the presented IPS is also improved.

The proposed method is open to use *crowd-sensing* [34], as it is possible to add knowledge without doing a new training. If there is just one user in the scenario, the system is able to update the RSSI centroids for each cell, using the new data obtained from the user (RSSI values and skeleton). The *crowd-sensing* technique continuously adjusts the parameters during system operation.

This method is also valid for identifying people in systems like OpenPTrack [3], where people is tracked over large areas in real time by means of multiple RGB-D sensors.

The system might be used in a museum to enhance the experience of the users. The work [35] makes use of the IPS technique presented in this article. Some panels of the museum might be interactive and would offer personalized information. In this field, future works will combine this IPS with a semantic database approach and analyze the complete pattern tracking of users’ movements in order to customize and enrich the user’s experience. 

## Figures and Tables

**Table 1:** Success rate according to the number of users and number of time stamps.

Number of Users	1	2	3	4	5	6	7	8	9	10
**1 time stamp**	100	82.24	56.53	30.89	15.04	5.32	3.64	0.94	0.50	0.50
**2 time stamps**	100	85.55	60.95	38.59	20.20	8.12	6.20	2.04	1.06	1.02
**3 time stamps**	100	90.37	72.87	52.85	32.35	17.14	14.66	5.64	2.86	2.98
**4 time stamps**	100	92.98	80.44	64.81	49.21	34.11	31.13	18.88	13.64	13.22
**5 time stamps**	100	95.10	86.30	73.03	59.31	45.45	44.89	31.79	24.86	25.59
**6 time stamps**	100	95.96	88.12	77.52	65.39	53.87	53.17	39.73	32.81	33.55
**7 time stamps**	100	97.80	94.36	87.92	80.72	73.07	72.01	61.55	57.69	56.87
**8 time stamps**	100	98.24	95.06	90.22	84.72	77.44	75.04	65.75	61.61	60.69
**9 time stamps**	100	99.59	98.74	97.54	96.00	94.14	93.70	91.04	89.48	88.96
**10 time stamps**	100	99.84	99.62	99.08	97.96	97.84	97.32	95.98	95.04	94.64

**Table 2:** Average distance error (meters) for different numbers of users and time stamps.

Number of Users	1	5	10	15	20
**1 time stamp**	0.2	1.73	1.99	2.00	2.00
**2 time stamps**	0.2	1.64	1.98	2.00	2.00
**3 time stamps**	0.2	1.42	1.95	2.00	2.00
**4 time stamps**	0.2	1.11	1.76	1.95	2.00
**5 time stamps**	0.2	0.93	1.54	1.80	1.99
**6 time stamps**	0.2	0.82	1.40	1.69	1.97
**7 time stamps**	0.2	0.55	0.98	1.28	1.77
**8 time stamps**	0.2	0.47	0.91	1.33	1.75
**9 time stamps**	0.2	0.27	0.40	0.54	0.85
**10 time stamps**	0.2	0.23	0.29	0.42	0.59
